# Progressive encephalomyelitis with rigidity and myoclonus: a pediatric case report and literature review

**DOI:** 10.1186/s12887-024-04899-5

**Published:** 2024-07-03

**Authors:** Yu Li, Jing-wen Wang, Qi-hui Chen, Ruo-hao Wu, Xiang-yang Luo, Zhan-wen He

**Affiliations:** 1 Department of Pediatrics, Sun Yat­sen Memorial Hospital, Sun Yat­sen University, Guangzhou, China; 2 Key Laboratory of Malignant Tumor Gene Regulation and Target Therapy of Guangdong Higher Education Institutes, Sun Yat-sen University, Guangzhou, China

**Keywords:** Autonomic dysfunction, Rigidity, Spasm, Pediatrics, Progressive encephalomyelitis with rigidity and myoclonus

## Abstract

**Background:**

Progressive encephalomyelitis with rigidity and myoclonus (PERM) is a rare and life-threatening autoimmune disease of the central nervous system. So far, only ten cases of PERM have been reported in children worldwide, including the one in this study.

**Case presentation:**

We report a case of an 11-year-old boy with PERM with an initial presentation of abdominal pain, skin itching, dysuria, urinary retention, truncal and limb rigidity, spasms of the trunk and limbs during sleep, deep and peripheral sensory disturbances, and dysphagia. A tissue-based assay using peripheral blood was positive, demonstrated by fluorescent staining of mouse cerebellar sections. He showed gradual and persistent clinical improvement after immunotherapy with intravenous immunoglobulin, steroids, plasmapheresis and rituximab.

**Conclusions:**

We summarized the diagnosis and treatment of a patient with PERM and performed a literature review of pediatric PERM to raise awareness among pediatric neurologists. A better comprehension of this disease is required to improve its early diagnosis, treatment, and prognosis.

## Background

Progressive encephalomyelitis with rigidity and myoclonus (PERM) is a rare and severe autoimmune inflammatory disease of the central nervous system, and it is classified as a type of stiff-person spectrum disorder (SPSD) [1]. PERM is rarer than the typical stiff-person syndrome, and it has more significant clinical manifestations, such as brainstem spinal cord, and autonomic dysfunctions [2–4]. PERM presents with varied initial symptoms and involves the extremities, brainstem, spinal cord, and autonomic nerves. Most patients have antibodies (such as GlyRAb, anti-amphiphysin, anti-GAD, and anti-DPPX). Moreover, PERM is closely associated with other autoimmune diseases such as thyroid disorders and diabetes. Most cases significantly improve with immunotherapy. In this study, the clinical manifestations and laboratory findings of a child with PERM were retrospectively analyzed. Furthermore, relevant literature was reviewed to deepen the clinicians’ understanding to enable early diagnosis and treatment and improve the prognosis of patients. The results of the study are reported below.

## Case presentation

The patient in this case was the first child of non-consanguineous parents. He had normal development, and no abnormalities were observed until he was 11 years of age. His family history was unremarkable. He was hospitalized for cervical lymphadenitis at the beginning of March 2022. During the antibiotic therapy with cephalosporin (ceftriaxone sodium, a total dose of 1 g/d for 7 days), he experienced abdominal pain, which was mainly in the stomach area. The abdominal pain was not relieved by normal treatment strategies. A few days later, he developed pruritus on anterior chest and abdomen. After symptomatic treatment, including acid suppression and antihistamines, the abdominal pain and pruritus improved but did not completely resolve. On April 10, 2022, his condition worsened. The patient initially presented with peripheral neuritis (abdominal pain and itchy skin) and gradually developed autonomic nervous system symptoms (dysuria and urinary retention) and spinal cord dysfunction (reduced symmetry of the Achilles-tendon and knee-jerk reflexes bilaterally), which were revealed by physical examination. Considering the manifestations of lymphadenitis at the early stages of the disease, we made a diagnosis of post-infection-induced immune-related inflammation of the nervous system. He was admitted to the neurology ward and was started on a course of immunoglobulin (IVIG, a total dose of 2 g/kg body weight per day was administered over 4 days), concomitant steroid therapy with dexamethasone (0.3–0.5 mg/kg), and pyridostigmine bromide (total dose: 30 mg/day). He subsequently developed altered consciousness and limb rigidity. Seizures could not be ruled out; therefore, antiepileptic drugs were added. After the commencement of oral antiepileptic therapy (e.g., valproate 24 mg/kg/day over 7 days and carbamazepine 100 mg/d for 7 days[increased to 200 mg/d over two weeks]) and intravenous midazolam, his limb stiffness temporarily subsided. His condition worsened, as he developed tachypnea and dysphagia. In addition to decreased hypoesthesia (involving superficial and deep sensations) below the umbilical level and the onset of bilateral leg weakness, deep sensory perception was also impaired, involving both Achilles- tendon areflexia and bilateral loss of the knee jerk. The dysesthesia and dyskinesia progressed over time. The patient was subsequently transferred to the pediatric intensive care unit. The patient was initially considered to have an immune disease. The patient’s condition continued to progress after the initiation of first-line immunotherapy (immunoglobulins and dexamethasone), with altered consciousness, limb stiffness, dysesthesia, and dyskinesia. We commenced plasmapheresis to rapidly relieve the patient’s clinical symptoms and reduce the pathogenic antibodies. Three sessions of plasmapheresis were performed. He regained consciousness within days, and the myoclonus ceased. The total duration of his ICU stay was seven days.

A second course of steroids and immunoglobulin therapy was administered. The gastric tube was removed due to the recovery of swallowing function. However, he relapsed and developed progressive dyskinesia. The child gradually developed upright head instability in addition to shrugging and neck turning weakness. The muscle strength of both lower limbs was 0, and the level of sensory impairment was up to the base of the neck. He developed generalized myoclonus during sleep. He also experienced sudden episodes of rigidity that lasted for tens of seconds. These symptoms were aggravated by emotional fluctuations. Those symptoms were similar to those of “stiff-man syndrome”. Simultaneously, he developed a disorder of consciousness characterized by a shallow state of coma. Stiff-person syndrome (SPS) should be considered when muscle stiffness and spasm involve the torso and lower extremities. PERM should be highly suspected when there are other cognitive, brainstem, and autonomic dysfunctions. We made a diagnosis of PERM. After the commencement of steroids and immunoglobulin therapy, the child’s initial symptoms of dyspnea, dysphagia, and abdominal pain were relieved. Considering the effectiveness of immunotherapy, the patient was started on the third session of immunoglobulins and dexamethasone. Rituximab treatment was initiated. Symptoms of rigidity and generalized myoclonus resolved. However, the sensory impairment below the neck was not relieved. After the fourth course of rituximab (one course every four weeks, 375 mg/m^2^ each) and symptomatic treatment, he progressively recovered motor function, gradually regained consciousness, and had resolution of sensory impairment. Oral low-dose methylprednisolone therapy was continued for six months after discharge. Only local mobility disorders of the lower limb from the hip to the ankle remained at the last follow-up.

The diagnostic workup included lumbar puncture, magnetic resonance imaging (MRI), electroencephalography (EEG), electromyography (EMG), screening for tumors, and a full antibody panel of serum and cerebrospinal fluid (CSF). The tests, including serum antigen and computed tomography imaging, were negative for tumors. Tests for serum and CSF antibodies (GlyR antibodies, GAD antibodies, DPPX antibodies, and NMDAR antibodies) were negative. A tissue-based assay (TBA) demonstrated positive cell-like fluorescence in neuronal dendrites of the hippocampus and Purkinje cells of the cerebellum. The serum antithyroid peroxidase antibody and antithyroglobulin antibody tests were positive. Repeated CSF analysis identified plasma cells and normal cells. Brain and spinal MRIs were normal. The electroencephalogram and the computed tomography imaging of the chest were normal. Electromyography revealed f waves over the ulnar, tibial, and right median nerves bilaterally.

## Discussion and conclusions

Whiteley et al. first described this disease in 1976 [5]. He reported two patients who presented with encephalomyelitis associated with subacute myoclonic spinal neuronitis and SPS. This condition was initially categorized as a stiff-person spectrum disorder (SPSD), but was later identified as PERM. PERM is an underrecognized and rare childhood disease. In 2013, Joana Damásio described the first pediatric case of PERM [6]. So far, only ten cases of PERM have been reported in children worldwide, including ours. PERM has insidious onset and various initial symptoms. It is rare and easy to misdiagnose. During the seven months of hospitalization, the patient we reported underwent lumbar puncture, electromyography, and imaging examinations several times. However, no definitive diagnosis was made until typical clinical manifestations appeared in the later stages of the disease. The whole diagnosis and treatment process is significant for reference purposes; therefore, we have reported this case to strengthen the understanding of this rare disease.

Diagnosing PERM is very challenging, and its diagnosis is primarily clinical. There are no internationally recognized guidelines, standards, or consensus on how to diagnose PERM. In 2022, Newsome and Johnson proposed the diagnostic criteria for all SPS-spectrum disorders, which include classic, partial, pure cerebellar ataxia, SPS-plus, and PERM. They also proposed examples of extended diagnostic criteria for PERM [7]:

A. Major criteria (1) clinical presentation involving typical body regions involved (neck, torso, extremities, brainstem and/or cerebellar symptoms); (2) hallmark triggers for spasms or increased rigidity (including abrupt loud noises, cold weather, open spaces, emotional stress (good and bad), and/or tactile stimuli); (3) examination findings, including an admixture of other phenotype findings plus encephalopathy, severe torso rigidity, and/or myoclonus (multifocal or generalized); (4) the presence of high titers of serum autoantibodies to the 65-kilodalton isoform of glutamic acid decarboxylase (GAD65), glycine receptor, or amphiphysin; (5) an EEG showing generalized slowing and/or epileptic discharges; and (6) the exclusion of alternative diagnoses and no better explanation for the syndrome;
B. Minor criteria (1) autonomic dysfunction; (2) the presence of CSF autoantibodies to GAD65, glycine receptor, or amphiphysin; (3) CSF pleocytosis; (4) CSF-restricted oligoclonal bands; (5) an EMG demonstrating co-contraction of agonist and antagonist muscles and/or continuous motor unit activity in the affected muscles (paraspinal or abdominal musculature and/or legs or arms); (6) a brain MRI demonstrating T2/contrast-enhancing lesion(s) in the brainstem; and (7) a brain fluorodeoxyglucose (FDG)-positron emission tomography demonstrating hyper-or-hypo-metabolism within the cortices.

PERM may be considered in any of the following situations: (1) Definitive diagnosis: meets all major and minor criteria; meets all major criteria and no minor criteria; meets four major criteria (which must include the presence of serum autoantibodies, the exclusion of alternative diagnoses, and no better explanation for the syndrome) and at least two minor criteria; or meets three major criteria (which must include the presence of serum autoantibodies, the exclusion of alternative diagnoses, and no better explanation for the syndrome) and at least two minor criteria. (2) Probable: meets three major criteria (which must include the exclusion of alternative diagnoses and no better explanation for the syndrome) and at least two minor criteria. (3) Possible: meets three major criteria (which must include the exclusion of alternative diagnoses and no better explanation for the syndrome) and less than two minor criteria, or meets two major criteria (which must include the exclusion of alternative diagnoses and no better explanation for the syndrome) and at least two minor criteria. Our patient had typical stiffness and spasms involving the torso and limbs. Emotional changes and tactile stimuli induced or aggravated the above symptoms. There were features of encephalopathy (such as altered consciousness), brainstem dysfunction (such as dysphagia and dyspnea), spinal cord dysfunction (such as hypoesthesia and itchy skin), and autonomic nervous system dysfunction (such as urinary retention). No other nervous system diseases or impairments in cognitive function could explain the stiffness and other symptoms in this patient. This case meets three major criteria and one minor criterion proposed by Newsome. It is also consistent in keeping with the characteristics and disease evolution pattern of PERM reported in the literature. Therefore, we made a diagnosis of PERM.

Common differential diagnoses of PERM syndrome include tetanus, psychogenic dyskinesia, and neuromyotonia (NMT). The typical symptom of tetanus is opisthotonos. It can be identified by the medical history, typical clinical manifestations, lack of response to benzodiazepines, and absence of relief during sleep. The main clinical features of psychogenic dyskinesia are sudden onset and sudden cessation. It can be identified by electromyography, and placebo treatment is effective. The main feature of neuromyotonia is muscle stiffness that is not relieved during sleep. It is also unresponsive to benzodiazepines.

The etiology of PERM is unknown, but it may be related to immunity. GlyRAb is believed to be associated with 70% of PERM cases. Other antibodies (including anti-amphiphysin [16%], anti-GAD, and anti-DPPX) are also associated with PERM, and 13% of cases are seronegative [8]. A recent retrospective analysis found that GlyR antibodies were associated with obvious brainstem, autonomic, and sensory symptoms. Patients with anti-GAD experience sporadic progressive ataxia, palatal myoclonus, and epilepsy. Patients with anti-DPPX have obvious eye movement disorders and gastrointestinal symptoms [9]. We performed a TBA using peripheral blood because our patient’s serum and CSF related antibodies (GlyR antibodies, DPPX antibodies, GAD antibodies, and NMDAR antibodies) tests were negative. Positive cell-like fluorescence was seen in the neuronal dendrites of the hippocampus and the Purkinje cells of the cerebellum. We could not complete the cell-based assay. Simultaneously, our patient simultaneously had antithyroid peroxidase antibodies, suggesting immune dysfunction involvement. Studies have found that PERM is closely associated with other autoimmune diseases such as thyroid disorders and diabetes [10]. Therefore, our patient requires monitoring for the development of potential autoimmune disease. Further investigation is needed to clarify the mechanism of interaction between these antibodies. Different immune specificities may indicate distinct underlying disease mechanisms and underpinnings which may explain some of the differences in clinical phenotype.

So far, only ten cases of PERM have been reported in children worldwide, including ours. We summarized the currently reported pediatric PERM cases in Table 1. We found that there were more female than male cases, and the cause of this discrepancy is unclear. The age of onset ranged from 14 months to 18 years, with an average of 14 years. Patients were generally older and had heterogeneous clinical features. All patients had an onset of stiffness and spasms. Brainstem symptoms were present in 78% of patients. Autonomic abnormalities and cognitive impairment occurred in 44% of patients.

**Table 1:** A literature review of pediatric PERM cases

Study	Damásio et al. [6]. 2013	Balint et al [14] 2014	Patel et al [15] 2015	Tchapyinikov and Borst [16] 2017	Yeshokumar et al [17] 2021	Yeshokumar et al [17] 2021	Yeshokumar et al [17] 2021	Liu. F et al [18] 2022	Samuel et al [19] 2023	Current case 2023
Sex, age (years)	Female, 1	Male, 15	Female, 15	Male, 18	Female, 15.5	Female, 8.4	Female, 14.8	Male, 4.5	Female, 16	Female, 11
Clinical features										
Initial presentation	Irritability, restless sleep, axial hyperextension, rigidity, and generalized myoclonus	Unsteadiness of the right arm, and excessive startling triggered by noise, or touching of the trunk	Muscle spasm triggered by doorbell rings	Urinary retention	Right knee pain or tightness, right-hand spasm, twisting of the right lower face, falls, and anxiety or depression	Seizures, progressive weakness that was more severe on the right than the left, fatigue, bilateral ptosis, and hyperlordosis.	Horizontal diplopia, upper back tightness and spasm, and left shoulder or arm stiffness	Vomit, soft legs, persistent right strabismus of both eyes, the disappearance of left forehead lines, closed eyes, and weakness in the nose	Hyperthermia, stiffness, shock, and respiratory distress	cervical lymphadenitis, abdominal pain, and skin itching
Late manifestation	Stiffness, spasm or myoclonus, brainstem signs, and dysautonomia	Stiffness, spasm or myoclonus, cognitive impairment, brainstem signs, and dysautonomia	Stiffness, spasm or myoclonus, cognitive impairment, and seizures	Stiffness, spasm or myoclonus, seizures, brainstem signs, and dysautonomia	Stiffness, spasm or myoclonus, and brainstem signs	Stiffness, spasm or myoclonus, seizure, and brainstem signs	Stiffness, spasm or myoclonus, and brainstem signs	Walking instability, diplopia, and limb shaking	Stiffness, spasm or myoclonus, cognitive impairment, seizures, brainstem signs, and dysautonomia	Stiffness, spasm or myoclonus, brainstem signs, and dysautonomia
Laboratory examination findings										
	CSF, MRI, EEG: normal; EMG: abnormal	CSF: lymphocytosis; MRI, EEG: normal; EMG; abnormal	CSF: oligoclonal band +; EEG: normal; MRI, EMG: abnormal	CSF: normal; MRI: abnormal; EEG, EMG: NA	CSF: oligoclonal band +; MRI, EEG: NA; EMG: abnormal	CSF: normal MRI, EEG: NA; EMG: abnormal	CSF, MRI, EEG: NA; EMG: normal	CSF, EMG: normal; MRI: NA; EEG: abnormal	CSF: Pleocytosis; MRI, EEG: abnormal; EMG: NA	CSF, MRI, EEG, EMG; normal
Antibodies										
	Serum/CSF GlyR: +/+	DPPX(+)	Serum/CSF GlyR: +/+ Other Abs: VGKC-complex Ab (+)	Serum/CSF GAD: -/NA	Serum/CSF GAD: +/NA	Serum/CSF GAD: +/+	Serum/CSF GAD: +/+	Serum/CSF GlyR: +/+	Serum/CSF GAD:+/NA	TBA:+
Treatment										
Symptomatic treatment	Benzodiazepines	Benzodiazepines	NA	Benzodiazepines, baclofen, and gabapentin	Benzodiazepines and baclofen	Benzodiazepines	Benzodiazepines and baclofen	Benzodiazepines	Benzodiazepines, baclofen, and gabapentin	Benzodiazepines and antiepileptics
Immunotherapy	Steroids and IVIg	Steroids, IVIg PLEX, and rituximab	None	Steroids, IVIg, and PLEX	Steroids, IVIg, PLEX, and rituximab	Steroids, IVIg, PLEX, and MMF	IVIg	Steroids, IVIg, and rituximab	Steroids, IVIg, PLEX, and rituximab	Steroids, IVIg, PLEX, and rituximab
Outcome and follow-up										
	Four relapses and mild speech delay	Recovery	Residual mild startle response and left hemiparesis	Residual clonus in the feet	MRS score of 3 on the most recent visit	MRS score of 3 on the most recent visit	MRS score of 4 at the most recent visit, and the patient is wheelchair bound.	walking has improved significantly, and the frequency and duration of excessive startle response and muscle rigidity episodes have significantly reduced.	Residual marked thoracolumbar scoliosis and hyperlordosis requiring surgery	Only a local mobility disorder of the lower limbs extending from the hip to the ankle remains.

MRS, Modified Rankin Scale; NA, not available; PLEX, plasma exchange

Although the incidence of dysautonomia is low, close monitoring is required because these patients may develop severe respiratory failure. Additionally, patients must be transferred to the intensive care unit for management. A quarter of these patients required tracheal intubation, and the mortality rate is as high as 40% [2]. We found GlyR antibodies, GAD antibodies, DPPX antibodies, and VGKC-complex antibodies in patients with PERM, which were similar to those in adult patients.

Currently, there is no consensus on the treatment recommendations for PERM, and the treatment mainly consists of immunotherapy and symptomatic treatment, similar to the treatment in adults [7, 8, 11, 12]. Newsome and Johnson proposed an upgraded approach to immunotherapy [7]: first-line: IVIG, corticosteroids, or plasma exchange; second-line: plasma exchange, rituximab, mycophenolate mofetil, azathioprine, or a combination of therapies; third-line: cyclophosphamide or a combination of therapies; and fourth-line: stem cell therapy. If the patient experiences acute exacerbations of symptoms during treatment, immunotherapy should be considered. If the patient does not respond to the above treatments, escalating to stronger immunotherapy should be considered. Commencing immunotherapy within two months of disease onset is recommended [8]. Benzodiazepines have been the cornerstone of the symptomatic treatment of SPSD because their main mechanism of action involves enhancing GABAergic pathways. Furthermore, they have muscle relaxation, anticonvulsant, and antianxiety effects. Our patient underwent midazolam therapy for the stiffness and spasms, with minimal improvement in symptoms. During this period, his condition progressed, with the onset of brain stem symptoms, difficulty swallowing, and difficulty breathing. Therefore we discontinued benzodiazepine therapy.

There are few studies on the long-term prognosis of PERM. 18% of patients with PERM experience a chronic disease course with acute exacerbations. Residual sequelae and recurrence are common. The time to relapse varies, ranging from a few weeks to nine years after onset [13]. Long-term follow-up is crucial for disease management. Our patient did not have any of the relevant antibodies that have been reported so far. He presented with acute disease and had periods of exacerbation. Treatment responses tended to fluctuate during the acute phase of the disease. He demonstrated a significant response to the combined escalation approach to immunotherapy, including the administration of IVIG, corticosteroids, plasma exchange, and rituximab. Subsequently, he progressively improved. Thus far, our patient has not experienced a disease recurrence. The reason for our patient’s poor response to initial treatment remains unclear. Some studies found that seronegative patients respond poorly response to treatment, with only 18% of them having a substantial response and 9% having a partial response. However, no recurrence occurred in this group of patients [8].

There were some shortcomings in the diagnosis and treatment of this child, including a lack of attention to trunk muscles, such as the paravertebral muscles, during several EMG examinations. Compared with the skeletal muscles of the limbs, the motor unit potential activity of the trunk muscles, especially those of the thoracolumbar and the rectus abdominis muscles, is more significant in patients with PERM. In addition, the patient’s condition fluctuated, starting with sensory disorders and symptoms of autonomic nervous involvement, followed by typical symptoms such as stiffness, muscle spasms, and features of brain stem and spinal cord dysfunction. This caused some confusion. The above factors delayed the diagnosis for this patient.

In conclusion, we summarized the diagnosis and treatment of a patient with PERM and also provided a summarized literature review of case reports on pediatric PERM to raise awareness among pediatric neurologists. A better comprehension of this disease is required to improve early diagnosis, treatment, and prognosis.

## Data Availability

Data is provided within the manuscript.

## Abbreviations

MRI: Magnetic resonance imaging
PERM: Progressive encephalomyelitis with rigidity and myoclonus
SPS: Stiff-person syndrome
SPSD: Stiff-person spectrum disorder
TBA: Tissue-based assay
